# Particle shape effect on heat transfer performance in an oscillating heat pipe

**DOI:** 10.1186/1556-276X-6-296

**Published:** 2011-04-05

**Authors:** Yulong Ji, Corey Wilson, Hsiu-hung Chen, Hongbin Ma

**Affiliations:** 1 Marine Engineering Department, Dalian Maritime University, Dalian 116026, People's Republic of China; 2 Department of Mechanical and Aerospace Engineering, University of Missouri, Columbia, MO 65211, USA

## Abstract

The effect of alumina nanoparticles on the heat transfer performance of an oscillating heat pipe (OHP) was investigated experimentally. A binary mixture of ethylene glycol (EG) and deionized water (50/50 by volume) was used as the base fluid for the OHP. Four types of nanoparticles with shapes of platelet, blade, cylinder, and brick were studied, respectively. Experimental results show that the alumina nanoparticles added in the OHP significantly affect the heat transfer performance and it depends on the particle shape and volume fraction. When the OHP was charged with EG and cylinder-like alumina nanoparticles, the OHP can achieve the best heat transfer performance among four types of particles investigated herein. In addition, even though previous research found that these alumina nanofluids were not beneficial in laminar or turbulent flow mode, they can enhance the heat transfer performance of an OHP.

## Introduction

Utilizing the thermal energy added on the oscillating heat pipe (OHP), the OHP can generate the oscillating motion, which can significantly increase the heat transport capability. Compared with the conventional heat pipe, the OHP has a number of unique features: (1) an OHP has a higher thermal efficiency because it can convert some thermal energy from the heat generating area into the kinetic energy of liquid plugs and vapor bubbles to initiate and sustain the oscillating motion; (2) the liquid flow does not interfere with the vapor flow because both phases flow in the same direction resulting in low pressure drops; (3) the structure of liquid plugs and vapor bubbles inside the capillary tube can significantly enhance evaporating and condensing heat transfer; (4) the oscillating motion in the capillary tube significantly enhances the forced convection in addition to the phase-change heat transfer; and (5) as the input power increases, the heat transport capability of an OHP dramatically increases. Because of these features, extensive investigations of OHPs [1-12] have been conducted since the first OHP developed by Akachi in 1990 [1]. These investigations have resulted in a better understanding of fluid flow and heat transfer mechanisms occurring in the OHP.

Most recently, it was found that when nanoparticles [13,14] were added into the base fluid in an OHP, the heat transport capability can be increased. The thermally excited oscillating motion in the OHP helps suspend some types of particles in the base fluid that would otherwise settle out of solution. Although nanoparticles added on the base fluid cannot greatly increase the thermal conductivity [14], the oscillating motion of particles in the fluid might have an additional contribution to the heat transfer enhancement beyond enhancing thermal conductivity. Ma et al. [13,14] charged the nanofluids (HPLC grade water and 1.0 vol.% diamond nanoparticles of 5-50 nm) into an OHP and found that the nanofluids significantly enhance the heat transport capability of the OHP. The investigated OHP charged with diamond nanofluids can reach a thermal resistance of 0.03°C/W at a power input of 336 W. Lin et al. [15] charged silver nanofluids with a diameter of 20 nm into an OHP and confirmed that the nanofluids can improve the heat transport capability of OHPs. With a filling ratio of 60%, their OHP can achieve a thermal resistance of 0.092°C/W. Qu et al. [16] conducted an investigation of the effect of spherical 56-nm alumina nanoparticles on the heat transport capability in an OHP, and found that the alumina particles can enhance heat transfer and there exists an optimal mass fraction. Although these investigations have demonstrated that the particles can enhance heat transfer in an OHP, it is not known whether there exists an optimum particle shape for a given type of particles.

In the current investigation, the particle shape effect on the heat transfer performance of an OHP was investigated experimentally. Ethylene glycol (EG) was used as the base fluid. Four types of nanoparticles with shapes of platelet (9 nm), blade (60 nm), cylinder (80 nm), and brick (40 nm) were studied to determine whether the optimum particle shape exists for the maximum heat transport capability of the OHP.

## Preparations and procedures of the experiment

**Figure 1:**
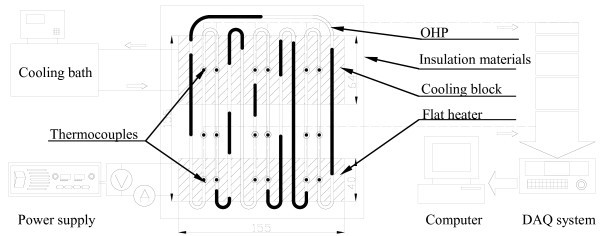
**Schematic of experimental system (units in mm)**.

The experimental system shown in Figure 1 consists of an OHP, circulator (Julabo-F34), cooling block, NI-DAQ system, power supply (Agilent-N5750A), and electrical flat heater. In order to form liquid plugs, a copper tube with an inner diameter of 1.65 mm and outer diameter of 3.18 mm was used for the OHP in the current investigation. As shown in Figure 1, the OHP has six turns and three sections: evaporator, condenser, and adiabatic section with the lengths of 40, 64, and 51 mm, respectively. The OHP was tested vertically, i.e., the evaporator on the bottom heated by a uniform electrical flat heater. The condenser section was directly attached to a cooling block which was cooled by a constant-temperature circulator. The data acquisition system controlled by a computer was used to record the experimental data. A total of 18 T-type thermocouples were placed on the outer surface of the OHP as shown in Figure 1 to measure the wall temperatures of the OHP. Figure 1 shows the locations of these thermocouples. The temperature measurement accuracy of the whole DAQ system is ± 0.25°C. The whole test section including the OHP, cooling block, and heater were well insulated to minimize the heat loss. Based on the insulation surface temperature, the power input uncertainty is less than 5% of the total power input.

### Nanofluids preparation

**Figure 2:**
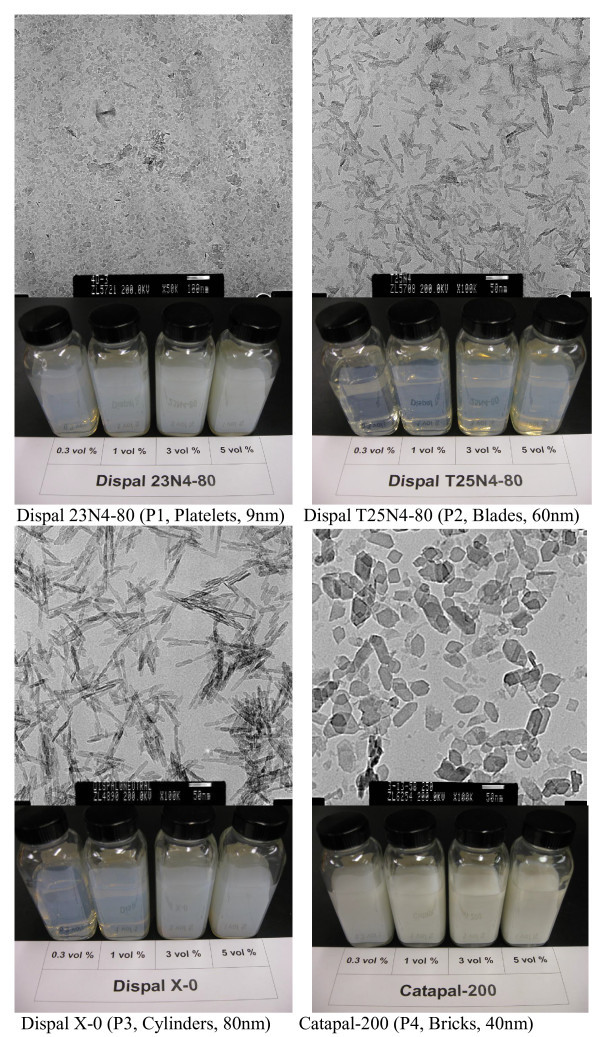
**TEM images of alumina nanoparticles (TEM images and designations provided by manufacturer) and photos of alumina nanofluids**.

For the current investigation, the nanoparticles of boehmite alumina with different shapes (platelet, blade, cylinder, and brick) were used. As shown in Figure 2, transmission electron microscopy (TEM: transmission electron microscopy) images were provided by the manufacturer (Sasol North America Inc.: Houston, Texas, U.S.) to determine the particle shape and size. EG 99+% (Fisher) and deionized water was mixed 50/50 by volume, and was used as the base fluid for all preparations. The particles were directly added into the base fluid at concentrations of 0.3, 1, 3, and 5 vol.%. As soon as the particles were added into the base fluid, the base fluid with particles was continuously mixed using a magnetic stirrer for 3 days. It was also sonicated with the ultrasonic oscillator for three 1-h sessions. Almost no sediments was observed a week after nanofluids preparation. Timofeeva et al. [17] studied the same nanofluids. The process of the nanofluids preparation was almost the same with the current investigation except that minor sediments were decanted a week after the nanofluid preparation in their work (maximum concentration change of 0.2 vol.%). The same nanoparticles and nanofluids were characterized carefully in [17] and the results showed that the crystallite sizes are close to particles size quoted by manufacturer, the alumina nanoparticles are composed of the same phase and mostly are single crystallites.

## Experimental procedures

Before the nanofluids were charged into the OHP, the base fluid (mixture of EG and deionized water 50/50 vol%) was charged into the OHP by the back-filling method [18]. All heat pipes were tested at a filling ratio of 50% in this paper. The OHP was tested vertically, i.e., the evaporator on the bottom and the condenser on the top. Prior to the test, the cooling bath (circulator) temperature was set at 20 or 60°C, which is defined as the operating temperature of the OHP. As soon as the cooling bath reached a temperature of 20 ± 0.3 or 60 ± 0.3°C, the power supply was switched on and the input power was added to the evaporator section of the OHP. The power was gradually increased in a step-wise mode with a power increment of 25 or 50 W depending on the total power. When the input power was less than 100 W, the increment was 25 W. When the input power was higher than 100 W, the increment was 50 W. When the input power was increased, the system needed time to reach a new steady state. The experimental data showed that when the power input was low, the time required to reach the steady state was about 30 min, and for a higher input power, it was about 10 min. When the evaporator average temperature changed less than 0.5°C within 1 min, it was defined that the test section reached steady state. The input power and the temperature data were then recorded by a computer. This was continued until the total power exceeded the 250 W limit of the heater used in the current investigation. Throughout the whole operating process, once the evaporator temperature exceeded 160°C, the test was stopped due to the temperature limit of the insulation materials. After the OHP charged with the base fluid was tested, the nanofluid of one shape particle with different volume fractions (0.3, 1, 3, 5 vol.%) were charged into the OHP and tested in the same way described above. It should be noted that a new OHP was manufactured for each nanoparticle shape and it was charged with the nanofluids from low volume fraction to high volume fraction to prevent nanoparticles left as residue inside the heat pipe from contaminating subsequent experiments.

Using the experimental setup and procedures described above, the effects of particle shape, particle volume fraction and operating temperature (20 and 60°C) on the heat transport capability in the OHP were studied. The evaporator temperature, *T*_e_, and the condenser temperature, *T*_c_, are based on the average temperature of six thermocouples placed on each of the evaporator and condenser sections, i.e., *T*_e _= ∑*T*_e*i*_/6 and *T*_c _= ∑*T*_c*i*_/6, respectively. The thermal resistance is defined as *R *= Δ*T*/*Q*, where Δ*T *is the temperature difference between evaporator and condenser and *Q *is the input power.

## Results and discussions

**Figure 3:**
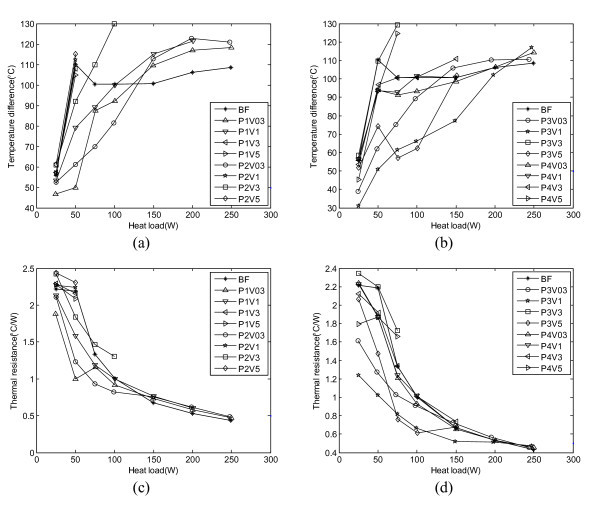
**Particle shape effect on (a), (b) temperature differenceand (c), (d) thermal resistance (operating temperature: 20°C, filling ratio: 50%, BF: base fluid, P1: platelet, P2: blade, P3: cylinder, and P4: brick)**.

**Figure 4:**
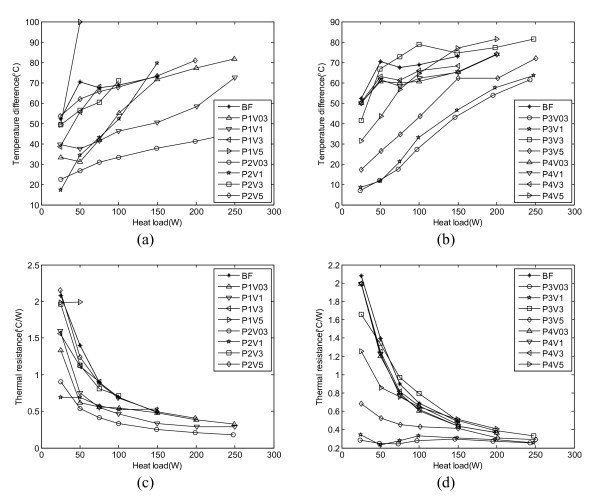
**Particle shape effect on (a), (b) temperature difference and (c), (d) thermal resistance (operating temperature: 60°C, filling ratio: 50%, BF: base fluid, P1: platelet, P2: blade, P3: cylinder, and P4: brick)**.

Figures 3 and 4 illustrate the particle shape effect on the OHP heat transfer performance at the operating temperature of 20 and 60°C respectively. In these figures, P1, P2, P3 and P4 stand for platelet-like, blade-like, cylinder-like, and brick-like shape particles, respectively, and V03, V1, V3, and V5 stand for the volume fraction of 0.3, 1, 3, and 5%, respectively. So, the combination of P and V can stand for different nanofluids. BF means the working fluid is the base fluid without any particles.

From Figure 3, it can be found that at the operating temperature of 20°C, the heat transport capability depends on the particle shape and volume fraction. When the input power is less than 100 W, the OHP charged with P1 (volume fraction < 3%), P2 (volume fraction < 1%), P3 (volume fraction < 3%), and P4 (volume fraction < 1%), respectively, can enhance the heat pipe performance. The heat transfer performance largely depends on the volume fraction. For the OHPs charged with P1, P2, and P4, respectively, the optimum volume fraction is about 0.3% while for the OHP charged with P3, the optimum fraction is about 1%. At a power input less than 100 W and a volume fraction of 0.3%, the OHP charged with P3 (cylinder) obtained the best heat transfer performance while the OHP charged with P4 (brick) showed the lowest among four types of particles. The sequence of heat transfer enhancement from the highest to lowest is: P3 (cylinder) > P2 (blade) > P1 (plate) > P4 (brick). However, when the input power is higher than 125 W, the OHP charged with P4 (brick) obtained the best heat transfer performance. The sequence of heat transfer enhancement from the highest to lowest becomes: P4 (brick) > P3 (cylinder) > P1 (plate) > P2 (blade).

From Figure 4, it can also be found that at the operating temperature of 60°C, the OHP heat transport capability depends on the particle shape and volume fraction. Almost all the nanofluids except P1V5 and P3V3 can enhance the heat transfer performance of the OHP. At a volume fraction of 0.3% and a power input less than 100 W, the sequence of heat transfer enhancement from the highest to lowest was: P3 (cylinder) > P2 (blade) > P1 (plate) > P4 (brick). But, as the input power increases, the sequence becomes: P2 (blade) > P3 (cylinder) > P4 (brick) > P1 (plate). It should be noted that the best volume fraction for all particles tested herein is 0.3%. From the results shown in Figures 3 and 4, it can be found that the operating temperature affects the heat transfer performance of the OHP as well. In previous work with these nanofluids [17], viscosity of the nanofluids decreases by at least half when the temperature increases from 20 to 60°C. This decreased viscosity significantly decreases the pressure drop, which can improve the oscillating motion in the OHP and therefore enhance the heat transfer performance of the OHP. This is one of those reasons why the operating temperature affects the heat transfer performance of the nanofluid OHP significantly.

**Figure 5:**
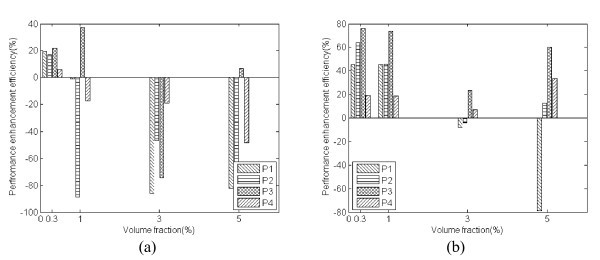
**Performance enhancement efficiency of nanofluid in an OHP at a filling ratio of 50% and an operating temperature of (a) 20°C and (b) 60°C**.

In order to evaluate the effect of nanoparticle shape on the heat transfer performance of nanofluids charged into a six-turn OHP in this investigation, the performance enhancement efficiency, *η*, is defined as follows:

where,  is the average thermal resistance of the OHP charged with base fluid, and  is the average thermal resistance of the OHP charged with nanofluid. Using the definition shown above, *η *can be determined as shown in Figure 5. It can be seen that at the volume fraction of 0.3%, all the nanofluids used in this study can enhance the heat transfer performance of the OHP. For other volume fractions, it largely depended on the operation temperature. At an operating temperature of 20°C, *η *tends to decrease as the volume fraction increases except cylinder-like particle (P3). The highest (37.3%) and lowest (-98.3%) values of *η *were found when the OHP was charged with P3V1 and P2V5, respectively. At an operating temperature of 60°C, all nanofluids except P1V3, P2V3, and P1V5 can enhance the heat transfer performance of the OHP. For blade-like particles (P2), cylinder-like particles (P3), and brick-like particles (P4), *η *decreases first and then increases as the volume fraction increases. For platelet-like particles (P1), *η *decreases as the volume fraction increases. When the OHP was charged with P3V03 and P1V5, the highest (75.8%) and lowest (-79.0%) values of *η *were found, respectively.

By comparing the current results (Figure 5) with the results obtained by Timofeeva et al. [17], it can be found that (1) while Timofeeva et al. [17] found that none of the nanofluids were beneficial in laminar or turbulent flow, these nanofluids in the current study enhanced the OHP performance and the performance was dependent on the particle shape and volume fraction; (2) while the cylinder-like particle (P3) is almost the worst particle in laminar and turbulent flow mode [17], it is the best particle in the current study; and (3) while as the volume fraction increases, the heat transfer performance of all nanofluids in laminar and turbulent flow tested by Timofeeva et al. [17] decreases, the results in the current study do not support these conclusions. For an OHP, the thermally excited oscillating motion of liquid plugs and vapor bubbles existing in an OHP is very different from the single phase flow investigated by Timofeeva et al. [17]. The oscillated nanoparticles in the OHP will directly affect the thermal and velocity boundary layers, which is very different from the one directional flow of laminar or turbulent flows. This might be the primary reason why the nanoparticles charged into an OHP can improve the heat transfer performance. However, the detailed mechanisms of heat transfer enhancement of these nanoparticles in an OHP are unclear and further research work is needed.

## Conclusions

The alumina nanoparticle shape effect on the heat transfer performance of an OHP was investigated experimentally and it is concluded that the alumina nanoparticles added in the OHP can enhance the heat transfer performance of OHP significantly and it depends on particle shape and volume fraction. For the six-turn OHP investigated herein, when the OHP was charged with EG and cylinder-like alumina nanoparticles, the OHP can achieve the best heat transfer performance among four types of particles, i.e., a performance enhancement efficiency, *η*, of 75.8% with an operating temperature of 60°C and volume fraction of 0.3%. In addition, it is demonstrated that the alumina nanofluids, which are not beneficial in laminar or turbulent flow mode, can enhance the heat transfer performance of the six-turn OHP investigated herein.

## Abbreviations

EG: ethylene glycol; OHP: oscillating heat pipe.

## Competing interests

The authors declare that they have no competing interests.

## Authors' contributions

YJ initiated the concept, developed the prototype, conducted the experiments and drafted the manuscript. CW participated in the oscillating heat pipe development and experimental setup. HC participated in the experimental investigation and data analysis. HM directed the prototype design, experiment, analysis and interpretation of experimental data, and participated in drafting and revising, and finalizing the manuscript. All authors read and approve the final manuscript.
